# Sufentanil sublingual tablet system versus oral oxycodone for management of postoperative pain in enhanced recovery after surgery pathway for total knee arthroplasty: a randomized controlled study

**DOI:** 10.1186/s40634-020-00306-x

**Published:** 2020-11-20

**Authors:** Emmanuel Noel, Luca Miglionico, Mickael Leclercq, Harold Jennart, Jean-François Fils, Nicolas Van Rompaey

**Affiliations:** 1 Anesthesiology, CHU Tivoli Hospital, Avenue Max Buset, 34, 7100 La Louvière, Belgium; 2 Orthopedic surgery, CHU Tivoli Hospital, La Louvière, Belgium; 3 ARS Statistica, statistics, Nivelles, Belgium

**Keywords:** Postoperative pain, Opioids, Multimodal analgesia, Fast track, Knee arthroplasty, Oxycodone, Sufentanil

## Abstract

**Purpose:**

Effectiveness of sufentanil sublingual tablet system (SSTS) compared to oral oxycodone in the management of postoperative pain after total knee arthroplasty (TKA) within an enhanced recovery after surgery (ERAS) protocol.

**Methods:**

This pragmatic, parallel, open label, randomized controlled, trial enrolled 72 adult patients scheduled for TKA under spinal anesthesia following ERAS pathway. In addition to multimodal analgesia, patients received SSTS 15 mcg (SSTS group) or oral oxycodone extended release 10 mg twice daily and oral oxycodone immediate-release 5 mg up to four times daily on demand (Oxy group) to control pain during 48 h postoperatively. The primary endpoint was pain measured using a numeric rating scale at 24 h postoperatively. Time to first mobilization, side effects and patient satisfaction were also recorded.

**Results:**

Median pain score at 24 h at rest was 3 [2–4] for Oxy group vs 2 [1.75–3] for SSTS group (*p* = 0.272) whereas median pain score on movement was 4 [3–6] vs 3 [2–5] respectively (*p* = 0.059). No difference in time to first mobilization was found between the two groups. The method of pain control was judged good/excellent for 83.9% of patients in the SSTS group compared with 52.9% in the Oxy group (*p* = 0.007). The incidence of nausea was 33% in SSTS group and 9% in Oxy group (*p* = 0.181).

**Conclusions:**

In complement to ERAS multimodal analgesia, sublingual sufentanil 15 mcg tablet system did not show clinically significant pain improvement compared to oral oxycodone after total knee arthroplasty.

**Trial registration:**

Clinical Trials: NCT04448457; retrospectively registered on June 24, 2020. https://clinicaltrials.gov/ct2/show/NCT04448457?cond=sublingual+sufentanil&cntry=BE&draw=2&rank=3

## Introduction

Total knee arthroplasty (TKA) is a common procedure expected to grow in the near future [24]. To cope with this increasing demand, optimization of hospitalized patient pathway without compromising quality of care is required. Enhanced Recovery After Surgery (ERAS) protocols, through multidisciplinary approach and protocol-driven pathways, meet these goals in major orthopedic surgeries, reflected by a decrease in length of stay, complications and better patient satisfaction [7, 11, 25, 27]. One of the major components of ERAS is early mobilization which relies on minimally invasive surgical techniques, short-acting anesthetics, prevention of nausea and pain control through multimodal analgesia [3, 10, 14]. Despite increasing knowledge about postoperative pain management, the use of nonsteroidal anti-inflammatory drugs (NSAIDs), corticosteroids and local infiltration analgesia (LIA) techniques, moderate to severe pain is still experienced by many patients after TKA and opioids are still necessary [1]. Intravenous (IV) opioids patient-controlled analgesia (PCA) is the gold standard for acute postoperative pain management but the need of an indwelling catheter is considered as a limiting factor for early ambulation [14, 20]. In this context, oral drug administration is privileged, when used as a part in a multimodal regimen, oral oxycodone demonstrated better pain relief over unimodal intravenous opioid [13].

Sufentanil is a potent synthetic μ receptor-specific agonist which is mainly used for intraoperative surgical analgesia. When given via sublingual route sufentanil shows a rapid onset due to high lipophilicity, a good bioavailability (60%) and a prolonged duration of action in comparison with IV sufentanil [4, 19]. Those characteristics make sublingual sufentanil a good candidate for postoperative pain treatment. The sufentanil sublingual tablet system (SSTS) (Zalviso®, Grünenthal GmbH, Aachen, Germany) is a new, pre-programmed, noninvasive, handheld system device for patient-controlled analgesia [4, 16]. This system combines the advantages of non-IV route and self-administration, which could fit patients’ needs in ERAS settings. The efficacy on postoperative pain relief has been shown in randomized controlled trials (RCT) for abdominal surgeries [16, 18, 21], plastic surgery [12] and major orthopedic surgeries [8, 16]. However, to our knowledge, use of SSTS in ERAS protocols has been reported only in few observational studies which conclude on the efficacy and the safety of the technique [23, 26, 29]. RCT comparing SSTS to an oral opioid effective regimen are also lacking.

Therefore, we conducted this trial to compare the efficacy of SSTS to oral oxycodone in the management of postoperative pain after TKA within an ERAS protocol. We hypothesized that SSTS could show a better profile on pain control, mobilization and patient satisfaction.

The primary endpoint of this study was to compare the effect of SSTS with oral oxycodone on pain intensity at 24 h postoperatively.

## Methods

### Study design

The study was a pragmatic, single-center, prospective, open label, randomized controlled trial conducted at CHU Tivoli Hospital, La Louvière, Belgium, to show the superiority of SSTS over oral oxycodone on pain control after total knee arthroplasty. The study design was approved by the Ethical Committee of Erasmus University Hospital, Brussels (ref P2017/348 on 21/06/2017). All patients signed an informed consent and the study was performed in accordance with the declaration of Helsinki. The study was registered retrospectively with ClinicalTrials.gov (NCT04448457). Inclusion criteria were adult patients (age ≥ 18 years) scheduled for unilateral total knee arthroplasty under spinal anesthesia, American Society of Anesthesiologists (ASA) class 1–3. Exclusion criteria were contraindications to our ERAS protocol, allergy to study medications, history of addiction or preoperative chronic use of opioids, unicompartmental or revision knee replacement.

### Perioperative management

All patients received oral midazolam, dosed at the discretion of the anesthesiologist, as anxiolytic premedication. Following the institutional ERAS protocol, 2 g cefazolin, 125 mg methylprednisolone and 1 g of tranexamic acid were administered intravenously 30 min prior to incision. Spinal anesthesia was performed with 8–12 mg of hyperbaric bupivacaine 0.5% without adjuvant. As part of the multimodal pain management protocol, all patients received by the surgeon local infiltration analgesia with 200 mL of ropivacaine 0,2% (with adjunction of epinephrine 2,5 mcg/mL). At the end of the surgery 3 g of tranexamic acid diluted in 70 mL of NaCl 0.9% were injected intraarticularly. All surgical procedures were performed by the same three surgeons without tourniquet and no drain was left in place.

At the arrival in post-anesthesia care unit (PACU), if necessary, pain was initially treated with IV piritramide until discharge criteria to the ward were met. For the postoperative multimodal pain management, application of ice pack on the wound area, acetaminophen 1 g PO four times daily and celecoxib 200 mg PO one time daily were given systematically to all patients. Postoperative nausea and vomiting (PONV) prophylaxis was administered according to institutional guidelines.

Patients followed our institutional rehabilitation protocol which consists of lifting the patient by the physiotherapist as soon as he returns from the recovery room, the patient is also seated whenever it’s possible on day 0 (day of surgery). On day 1, the patient starts passive motion machine (Kinetec®, Tournes, France), exercises in the physiotherapy room, occupational therapy program, and so on, on day 2.

### Randomization and intervention

Patients were randomly assigned to study groups (allocation ratio 1:1) in fixed blocks of 4 using computer-generated random numbers kept in sealed envelopes. Envelope was opened at the arrival of the patient in the operating theater and subjects were allocated to the sublingual sufentanil tablet system (SSTS) group or the oral oxycodone (Oxy) group.

Patients enrolled in the SSTS group were instructed for the use of the device in PACU and the first dose was administered there allowing sublingual administration of sufentanil 15 mcg tablet with a fixed 20-min lockout interval. In the Oxy group, patients received oral oxycodone extended-release (ER) 10 mg twice daily and oral oxycodone immediate-release (IR) 5 mg up to four times daily on demand when numeric rating scale (NRS) > 3. In case of insufficient pain management, patients could receive rescue intramuscular morphine injection and patients would be automatically excluded from the study.

### Outcomes assessment

Primary endpoint was pain score at 24 h postoperatively. Pain was assessed using a verbal 11-point numeric rating scale (NRS-11) ranging from 0 (“No pain”) to 10 (“Worst imaginable pain”). The verbal NRS-11 is a well validated, reliable and sensitive tool to evaluate acute postoperative pain [2, 6]. Patients were asked to evaluate their pain at rest (PAR) and pain evoked by passive or active flexion of the knee (movement evoked pain - MEP). Secondary outcomes were pain scores measured 2 h, 6 h after surgery and every 6 h thereafter up to 48 h, success of first mobilization (with or without aid) assessed by the first upright standing position followed by success of mobilization at these different timepoints, nausea and vomiting and finally satisfaction for the method of pain control assessed using Patient Global Assessment (PGA) of the method of pain control questionnaire at the completion of the 48 h study period which consist on a 4-point categorical scale, where 1 = poor, 2 = fair, 3 = good and 4 = excellent [22].

### Statistical analysis

Sample size calculation was estimated on the basis of an absolute reduction of NRS score in SSTS group compared to Oxy group. Analysis of pain scores obtained before the start of the study on 7 patients treated by oxycodone indicated a mean pain score of 5.587 with a standard deviation of 1.46 at 24 h after surgery. In order to show a difference of 2 points of NRS score with a power of 0.9 and a bilateral alpha risk of 0.05 we calculate the need of 28 patients per group, considering a minimal clinically important difference of 1. Taking into account an estimated drop-off of 20%, we conclude that a total of 70 patients were needed. We used the ‘Trial Size’ package of the R software to perform the sample size calculation.

Continuous data were compared by means of T-test when homogeneity of variances, tested with the Bartlett’s test, and normality of the residuals, tested with the Shapiro-Wilks test, were reached and means and standard deviations (SD) are reported. When homogeneity of the variance or normality of the residuals were not proved, Wilcoxon signed rank test was performed on rank data and medians and interquartile ranges ([Q_25_–Q_75_]) are reported. For count data, the Pearson Chi-Squared test was performed to compare proportions. For time to event data, Kaplan–Meier product-limit estimators of cumulative rates of patients reaching the event (success of first mobilization) at follow-up time points were calculated. A log-rank test was used to compare the two treatment groups. We used the software R, version 3.4.3 (R Core Team, 2017) to perform the statistical analyses. Prism version 8.4.2 (GraphPad Software, Inc.) was used to construct the figures.

## Results

**Fig. 1:**
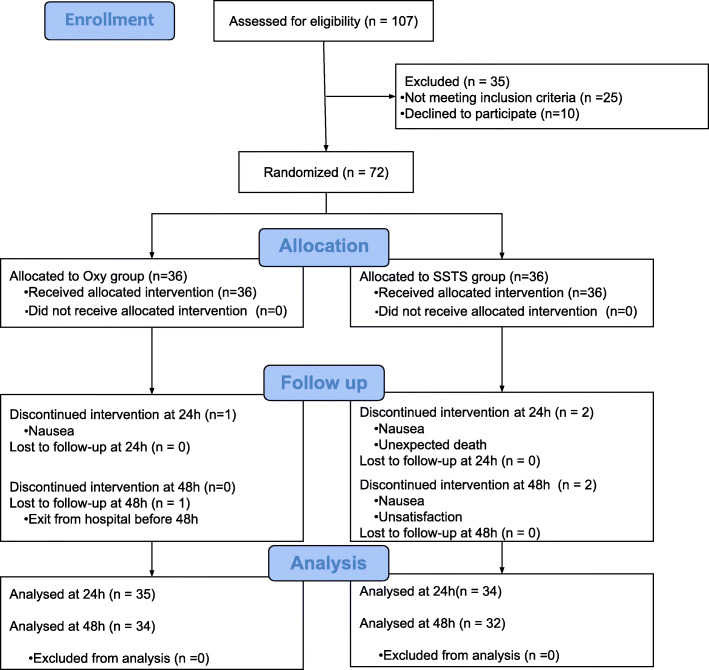
Participant Flow (CONSORT diagram). SSTS = sufentanil sublingual tablet system

**Fig. 2:**
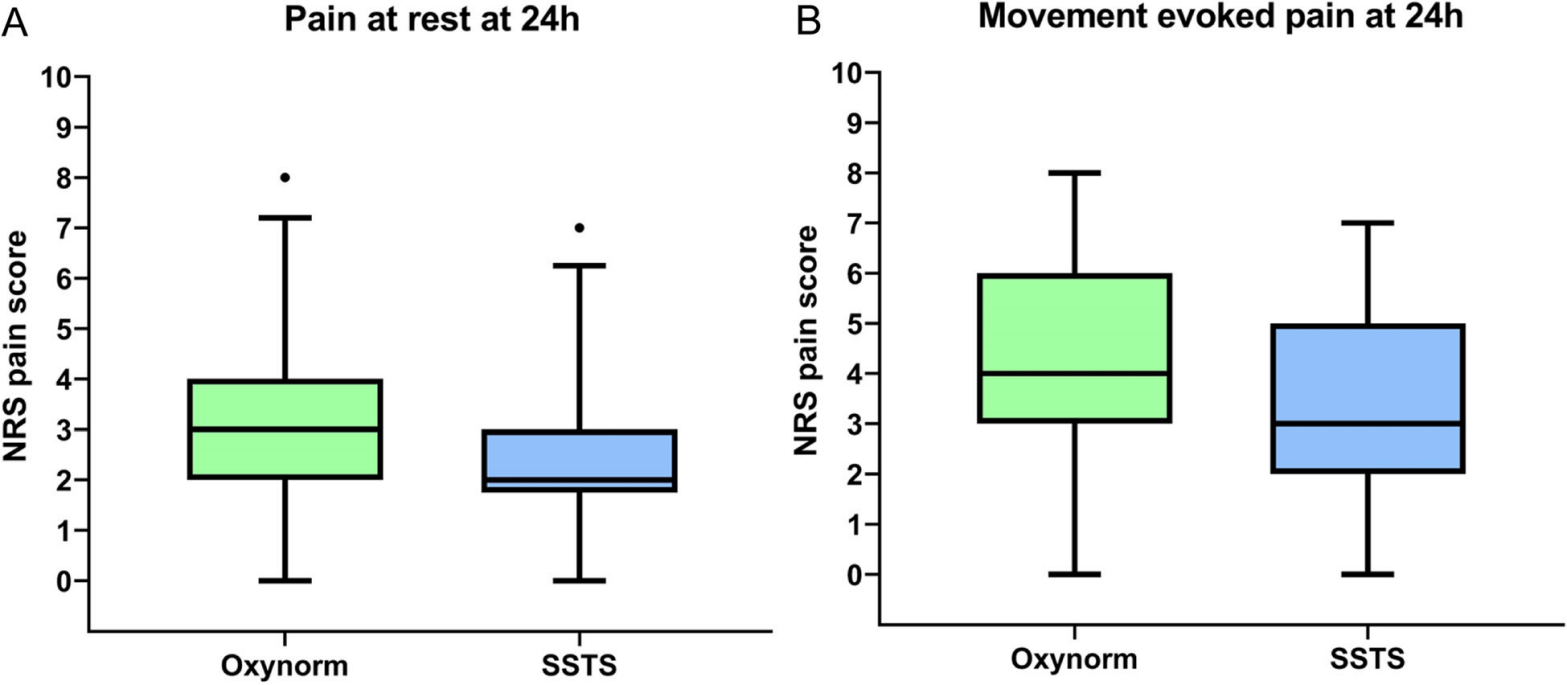
Box and whisker plots of postoperative pain score at 24 h **a**. Pain score at rest. **b**. Dynamic pain score. Solid horizontal lines represent the median, boxes indicate interquartile range (Q25-Q75), whiskers represent 5th percentile and 95th percentile and dots are the extreme values. No statistical difference was found

**Fig. 3:**
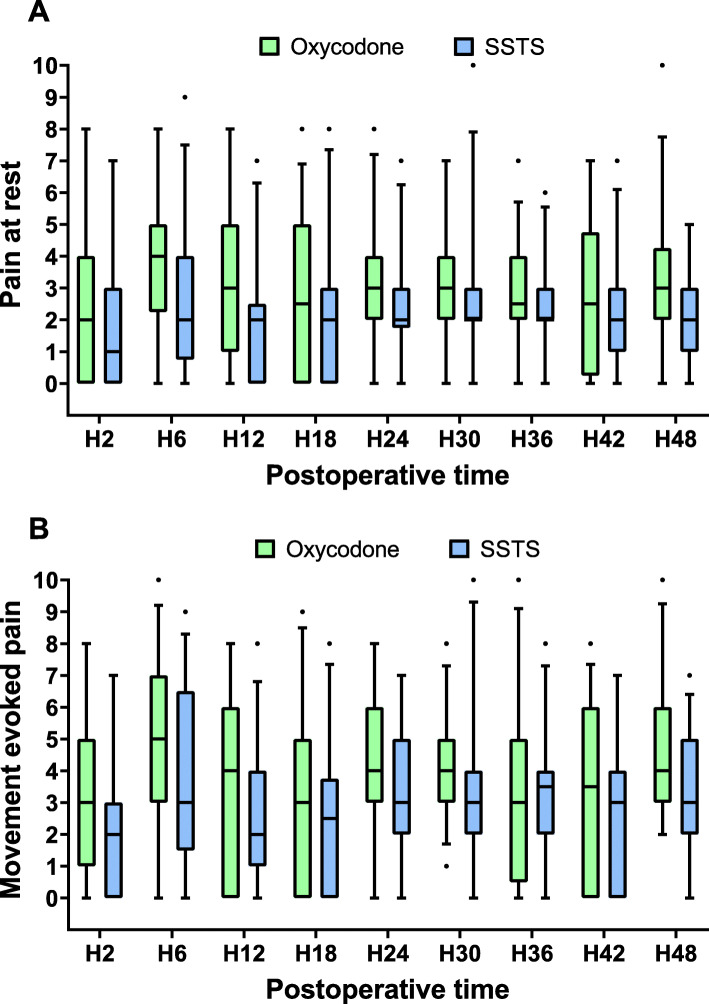
Box and whisker plots of postoperative pain scores over 48 h. **a**. Pain score at rest. **b**. Dynamic pain score. Solid horizontal lines represent the median, boxes indicate interquartile range (Q25-Q75), whiskers represent 5th percentile and 95th percentile and dots are the extreme values. No statistical difference was found. SSTS = sufentanil sublingual tablet system

**Fig. 4:**
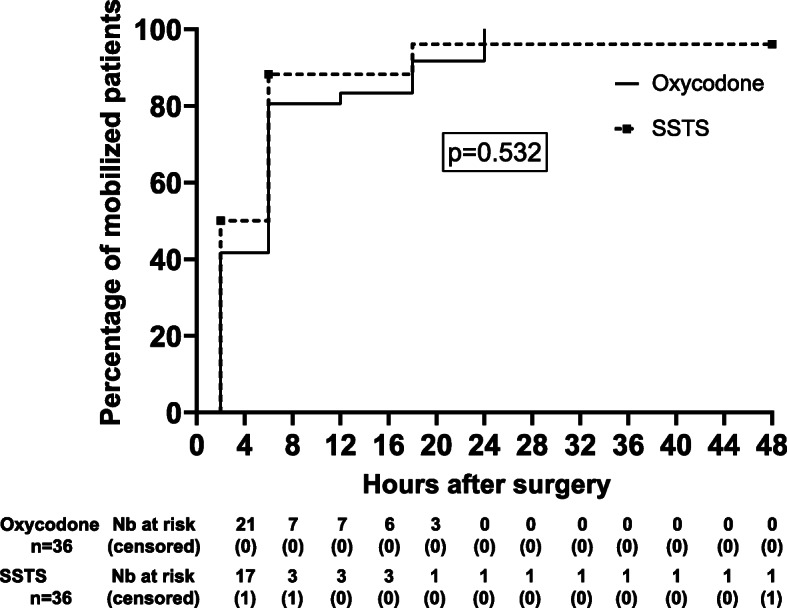
Kaplan–Meier cumulative event rates for time to first mobilization. Solid square indicates censored patient. No statistical difference was found. SSTS = sufentanil sublingual tablet system

**Fig. 5:**
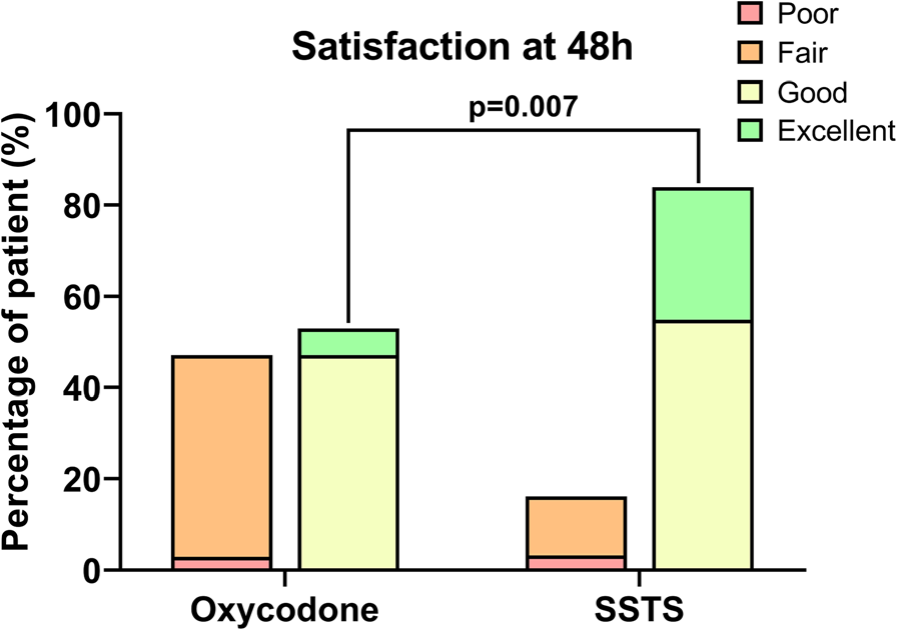
Patient Satisfaction

One hundred-seven patients were screened for eligibility between September 2017 and July 2018. Seventy-two patients were recruited and prospectively randomized between the two groups, 69 patients completed the follow-up at 24 h and 66 patients at 48 h. Three patients stopped prematurely the study due to nausea discomfort, one was excluded due to pain control insufficiency, one asked to leave the hospital at day 1 and one patient died in the afternoon at day 0 (Fig. 1). Because no relationship was found between the use of medications and the death, the Ethical Committee gave his approval to continue the study. There were no differences in the baseline characteristics between the two groups (Table 1). Median piritramide consumption in the PACU was 0 [0–2.75] for Oxy group vs 0 [0–0] for SSTS group (*p* = 0.239). During the 48 h observation period, all patients in the Oxy group received 50 mg of oxycodone ER, the mean oxycodone IR consumption was 33.6 (7.8) mg. In the SSTS group, the mean tablet consumption was 14.4 (7.84) tablets or 215.4 (117.6) mcg of sufentanil. Concerning the primary endpoint, pain score at 24 h, there was no statistical difference found between the two groups. Median pain score at rest was 3 [2-4] Oxy group vs 2 [1.75–3] for SSTS group (*p* = 0.272) whereas median dynamic pain score was 4 [3-6] vs 3 [2-5] without hyperlink respectively (*p* = 0.059) (Fig. 2). Nine patients (25.7%) described moderate-to-severe pain (NRS ≥ 4) in Oxy Group vs five patients (14.7%) (*p* = 0.255) in SSTS group at rest, and 23 (65.7%) vs 15 (44.1%) (*p* = 0.071) respectively on movement. No statistical differences were found neither in static nor in dynamic pain over the 48 h postoperative period (Fig. 3). Seven patients (9%) complained of nausea in the Oxy group and 12 patients (33%) in the SSTS group (RR 0.58, 95% confidence interval [0.260 to 1.270], *p* = 0.181). Three (8%) patients suffered vomiting in the Oxy group and 4 (11%) in the SSTS group (*p* = 0.690). One patient complained of pruritus in the Oxy group. As shown in Fig. 4, time to first mobilization was not different between groups. At the end of the study, the assessment of the method of pain control was judged Good/Excellent for 26/31 (83.9%) patients in the SSTS group compared with 18/34 (52.9%) in the Oxy group (*p* = 0.007) (Fig. 5).

**Table 1:** Patients’ characteristics

	Oxy group (*n* = 35)	SSTS group (*n* = 34)	*p*-value
Age (years)	65.3 ± 9.0	67.6 ± 8.2	0.262
Sex (woman)	20 (57.1%)	21 (61.7%)	0.807
ASA status (1/2/3)	9/22/4	9/23/2	–
Weight (kg)	90 [78–100]	87.5 [79.75–98]	0.874
Height (cm)	166.6 ± 9.3	166.4 ± 9.6	0.868
BMI (kg/m^2^)	32.11 ± 6.5	32.6 ± 5.5	0.733

Values are expressed as mean ± standard deviation, median with interquartile range [Q25-Q75] or number and percentage

*BMI* Body Mass Index, *SSTS* sufentanil sublingual tablet system

## Discussion

In our pragmatic prospective randomized study, SSTS 15 mcg showed no clinical benefit compared to a conventional oxycodone-based treatment for postoperative pain management in a population of TKA patients managed with our ERAS protocol. The lack of meaningful effect on pain score was noted for pain at rest but also for movement-evoked pain, a critically important outcome measure especially in the context of TKA [5]. Our data underlines the fact that, even with multimodal analgesia, TKA remains a painful procedure as seen in other studies [9]. While SSTS 15 mcg is indicated for the management of moderate to severe acute postoperative pain, SSTS treated patients showed no significant pain improvement and almost 50% of the patients are still experiencing NRS ≥ 4 on movement. Moreover, only one patient needed rescue analgesia and he was treated in the SSTS group.

Comparison to other studies is limited because few RCT addressed specifically the role of SSTS on postoperative pain. Compared to placebo without any co-analgesics, SSTS 15 mcg and 20 min lockout interval improved mean [SEM] summed pain intensity difference (SPID) from baseline over 48 h (SSTS 105.6 [10.14] vs placebo 55.58 [13.11]) after open abdominal surgeries [21]. Similar effects were observed in orthopedic surgeries where Jove et al. [8] showed that STSS 15 mcg could improve SPID 48 h after TKA or total hip arthroplasty (SSTS 76 [7] vs placebo − 11 [11]). In this study, mean NRS pain scores at 24 h were 3.9 (±0.2) for SSTS versus 5.1 (±0.4) for placebo (*p* = 0.002), one could argue on the clinical relevance of this result. The lack of multimodal analgesia as well as the absence of an effective control group could explain the pronounced effect of SSTS in these studies. This hypothesis is supported by the lack of difference on postoperative pain scores when SSTS was compared with continuous morphine after cardiac surgery [28]. After major orthopedic surgeries, no differences between SPID over 24, 48 or 72 h was seen when compared to intravenous morphine PCA for the management of acute postoperative pain although faster analgesia was observed in the first 4 h [16]. Because IV PCA restricts mobility, we decided to use as active comparator oral oxycodone treatment. SSTS and oral oxycodone have approximately the same onset (15–30 min) [18, 21] but SSTS does not expose patients to prolonged analgesic gaps due to absence of involvement of nurses in analgesic administration. Lack of gap and better titration should optimize pain control as seen with other PCA systems as suggested previously [23]. This optimized analgesia was not reflected in our study neither in a retrospective trial on 227 patients operated for TKA in an ERAS environment [29]. Similarly, to our study, all patients received ropivacaine LIA, acetaminophen and NSAIDS (metamizole three times daily) for multimodal analgesia. SSTS 15 mcg treated patients (*n* = 72) were compared to those who received oral oxycodone (*n* = 68) or oral oxycodone with dexamethasone given preoperatively (*n* = 87). Lowest and highest NRS pain scores at rest were compared during the first postoperative 48 h but SSTS showed no improvement on pain scores. Of note, sufentanil consumption was in the same range observed in our study (mean 13.75 (11.96) tablets over 48 h).

In our ERAS pathway, more than 80% patients in both groups were mobilized on day 0. With this high success of first mobilization the role of SSTS in functional outcome as early mobilization seems to be limited.

Incidence of adverse events was limited, but 33% of patients for SSTS group versus 9% of patients for Oxy group reported nausea during the 48 h which is in accordance with the literature [4, 15]. Even if this difference was not significant, which could be explained by the small sample size, nausea was the main reason for dropping out the study and should be taken into consideration. Although studies showed that SSTS could save time at the ward [17, 26, 29], the most interesting advantage from the use of SSTS based on our study was the patient’s satisfaction rate, as mentioned in other studies [5, 16]. This satisfaction could be explained by the easy use of this new PCA device and the feeling of personalization due to the thumb tag, but a Hawthorne effect is probably involved because of the open blind design and the novelty of the device.

Concerning the use of the system, we found some issues with the operation of the device, we noticed always the same problem: the thumb tag had to be replaced for three patients, especially at the beginning of the study.

Regarding the limited efficacy of the system and the high prevalence of nausea, the economic burden seems not to be justified even if patient satisfaction is improved. Indeed, in our hospital, the cost of one tablet of Oxynorm IR is €0.085, Oxynorm ER is €0.125 and the cost of the 40 tablets cartridge is €105. Regarding the mean consumption in each group, the total cost over 48 h is €1.195 for Oxy group and €105 for SSTS group. The price of the device (around €1400) as well the cost of the thumb patch should also be taken into account. Our study had several limitations. The use of mean NRS at 24 h as the primary endpoint may be less sensitive than SPID to detect a difference between treatment. The open label design could have influenced the PGA as explained above.

## Conclusion

In complement to ERAS multimodal analgesia, sublingual sufentanil 15 mcg tablet system did not show significant pain improvement compared to oral oxycodone after total knee arthroplasty.

## Data Availability

The datasets used and/or analyzed during the current study are available from the corresponding author on reasonable request.
